# Cancer-derived C-terminus-extended p53 mutation confers dominant-negative effect on its wild-type counterpart

**DOI:** 10.1093/jmcb/mjab078

**Published:** 2021-12-16

**Authors:** Shibo Huang, Bo Cao, Jieqiong Wang, Yiwei Zhang, Elisa Ledet, Oliver Sartor, Yuqin Xiong, Shelya X Zeng, Hua Lu

**Affiliations:** Institute of Clinical Pharmacology, Nanchang University, Nanchang 330006, China; Department of Biochemistry & Molecular Biology, Tulane University School of Medicine, New Orleans, LA 70112, USA; Tulane Cancer Center, Tulane University School of Medicine, New Orleans, LA 70112, USA; Department of Biochemistry & Molecular Biology, Tulane University School of Medicine, New Orleans, LA 70112, USA; Tulane Cancer Center, Tulane University School of Medicine, New Orleans, LA 70112, USA; College of Pharmacy, Xavier University of Louisiana, New Orleans, LA 70125, USA; Department of Biochemistry & Molecular Biology, Tulane University School of Medicine, New Orleans, LA 70112, USA; Tulane Cancer Center, Tulane University School of Medicine, New Orleans, LA 70112, USA; Department of Biochemistry & Molecular Biology, Tulane University School of Medicine, New Orleans, LA 70112, USA; Tulane Cancer Center, Tulane University School of Medicine, New Orleans, LA 70112, USA; Tulane Cancer Center, Tulane University School of Medicine, New Orleans, LA 70112, USA; Tulane Cancer Center, Tulane University School of Medicine, New Orleans, LA 70112, USA; Institute of Clinical Pharmacology, Nanchang University, Nanchang 330006, China; Department of Biochemistry & Molecular Biology, Tulane University School of Medicine, New Orleans, LA 70112, USA; Tulane Cancer Center, Tulane University School of Medicine, New Orleans, LA 70112, USA; Department of Biochemistry & Molecular Biology, Tulane University School of Medicine, New Orleans, LA 70112, USA; Tulane Cancer Center, Tulane University School of Medicine, New Orleans, LA 70112, USA

**Keywords:** mutant p53-393*78, mutant p53-374*48, p53, longer C-terminus p53, dominant-negative effect, drug resistance, acetylation

## Abstract

The vast majority of p53 missense mutants lose the wild-type (wt) function and/or exert ‘dominant-negative’ effects on their wt counterpart. Here, we identify a novel form of p53 mutation with an extended C-terminus (p53 long C-terminus, p53LC) in a variety of human cancers. Interestingly, the two representative mutants (named ‘p53-374*48’ and ‘p53-393*78’) as tested in this study show both loss-of-function and dominant-negative phenotypes in cell proliferation and colony formation assays. Mechanistically, p53LCs interact with and retain wt p53 in the cytoplasm and prevent it from binding to the promoters of target genes, consequently inhibiting its transcriptional activity. Also, p53LCs are very stable, though not acetylated in cells. Remarkably, the p53LCs can desensitize wt p53-containing cancer cells to p53-activating agents. Together, our results unveil a longer form of p53 mutant that possesses a dominant-negative effect on its wt counterpart, besides losing its wt activity.

## Introduction

The tumor suppressor p53 is the most important genome guardian, as it activates the expression of a multitude of genes responsible for cell-cycle control, DNA repair, senescence, ferroptosis, autophagy, and apoptosis in response to a variety of stressors (Zhou et al., 2018). It is the most mutated gene or inactivated protein in all types of human cancers, as ∼50% of human tumors harbor mutated TP53 (Pfister and Prives, 2017). Wild-type (wt) p53 protein is composed of an N-terminal transactivational domain (TAD), a central DNA-binding domain (DBD), a tetramerization domain, and a C-terminal regulatory region (Zhou et al., 2018). Among the p53 mutations, >74% are missense mutations (Zhou et al., 2018). Approximately 80% of the missense mutations occur in the p53 DBD, which is essential for sequence-specific DNA binding. Several hotspot mutations in this domain, such as R175, R248, R249, and R273, are identified in various primary and metastatic human cancers (Baugh et al., 2018; Tang et al., 2020). In addition to loss of function (LOF), these mutant p53s can execute their dominant-negative (DN) effects on the wt p53 activity and also possess gain of a new oncogenic function (GOF), leading to malignancy (Silva et al., 2013).

The p53 protein functions as a homotetrameric transcription factor in the nucleus (Shaulsky et al., 1990; Joerger and Fersht, 2016). Cytoplasmic sequestration and defective translocation of p53 have been suggested as the mechanisms of underlying p53 inactivation (Keshelava et al., 2001). The C-terminus of p53 contains a cluster of several nuclear localization signals (NLSs) that mediate its import into the nucleus (Shaulsky et al., 1990). Some mutant p53s could not be transported to the nucleus (Shaulsky et al., 1990; Liao et al., 2017). Posttranslational modifications (PTMs), such as acetylation, can often enhance the transcriptional activity of p53 by stabilizing and/or activating this protein in the nucleus (Gu and Roeder, 1997; Liu et al., 2019). For instance, several C-terminal lysine residues of p53 (K372, K373, K381, K382, and K386) are acetylated by p300 and CBP, leading to activation of the transcriptional activity of p53 (Avantaggiati et al., 1997; Gu and Roeder, 1997; Liu et al., 2019).

The p53 stability is regulated by MDM2 via ubiquitination-dependent proteolysis in a negative-feedback fashion (Vousden and Lu, 2002; Michael and Oren, 2003; Levine, 2020). Ubiquitin can be interlinked via any of its lysines (such as K48 and K63) and through the amino terminal methionine (Hock and Vousden, 2014). Among these lysines, K48 is used for forming ubiquitin chains that lead to proteasomal degradation, whereas K63 is for regulation of protein functions or signaling (Kulathu and Komander, 2012). Besides regulating p53 stability, MDM2 also inhibits p53 transcriptional activity. However, mutant p53s are often quite stable and accumulate to high levels in tumor cells due to lack of this MDM2 negative-feedback regulation (Lukashchuk and Vousden, 2007).

In this study, we report a novel form of p53 mutation with an extended C-terminus, named ‘p53LC’, identified in various cancers. The starting point of the extension is either within the extreme C-terminus or at the end of the p53 protein due to open reading frame (ORF) shift. Our further study of two representative p53LCs demonstrates that they not only possess an LOF phenotype owing to their impaired nuclear localization and DNA-binding activity, but also exhibit DN effects on wt p53. The p53LCs can desensitize wt p53-harboring tumor cells to the treatment with actinomycin D or nutlin-3. These findings are clinically relevant, as this type of mutation is largely heterozygous with an allele of wt TP53 in cancer patients.

## Results

### p53LCs lose wt p53 activity

**Figure 1:**
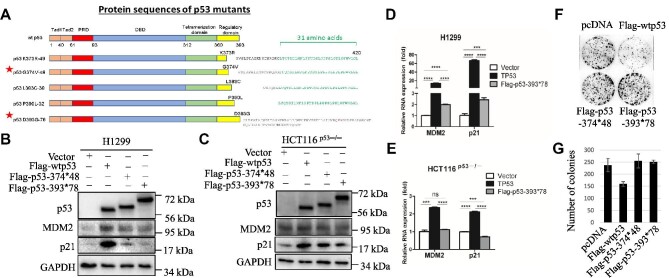
p53LCs display loss of wt p53 functions. (**A**) The schematic of the functional domains and mutations of the C-terminus-extended p53s. (**B** and **C**) H1299 or HCT116^p53−/−^ cells were transfected with the vector, wt p53, p53-374*48, or p53-393*78 plasmid and harvested 48 h after transfection for IB analysis with indicated antibodies. (**D** and **E**) H1299 or HCT116^p53−/−^ cells were transfected with the vector, wt p53, or p53-393*78 plasmid and harvested 48 h after transfection for RT–qPCR analysis. (**F** and **G**) H1299 cells transfected with plasmids as indicated were seeded for colony formation assays. (**F**) Cell colonies were fixed and stained with crystal violet solution. (**G**) The number of colonies was quantified. Data are presented as mean ± SEM. of triplicate experiments. *P < 0.05 or **P < 0.01 was determined by a two-tailed *t*-test.

The idea to examine the role of the p53LCs in cancer cell survival and proliferation stemmed from our initial observation of a germline p53 mutation in a man with an independent history of prostate, renal, thyroid, and lung cancers. This mutant was created due to missing G at the codon for amino acid (aa) 374 in the C-terminus of p53. This ‘loss-of-G’ mutation caused an ORF shift, resulting in a longer p53 with an extended C-terminus consisting of extra 48 amino acids that are different from wt p53 (Figure 1A). This prompted us to analyze the cBioportal database (Cerami et al., 2012; Gao et al., 2013). Strikingly, we found multiple similar p53 mutants, i.e. with C-terminus extension, present in a variety of cancer types, including lung, colorectal, prostate, and breast cancers (Supplementary Figure S1). Comparing these mutants in detail revealed two forms of p53’s C-terminal extension (Figure 1A): (i) one occurred at aa 373 or aa 374 of p53 due to the ORF shift at this position as a result of loss of G, resulting in a longer p53 with extra 48 amino acids at its C-terminus, sharing the same last 31 amino acids, named as ‘p53-374*48’; (ii) the other occurred at the end of p53 due to addition of G to the codon for aa 393, bringing about one ORF shift and consequently a much longer and different polypeptide named ‘p53-393*78’. Thus, we focused on studying p53-374*48 and p53-393*78 (of note, the popular p53-382*40 was not chosen because this mutant was not shown in our initial search).

To determine whether these two p53LCs might still possess wt p53 activity, we transfected H1299 (p53-null) and HCT116^p53^^−^^/^^−^ cells with Flag-wtp53, Flag-p53-393*78, or Flag-p53-374*48 alone and assessed their ability to induce the expression of p53 target genes, such as MDM2 and p21, by reverse transcription–quantitative polymerase chain reaction (RT–qPCR) and immunoblotting (IB) analyses. Even though the p53LCs have intact and mutation-free N-terminal TAD and central DBD, they failed to induce the expression of these genes to different degrees in the two cell lines (Figure 1B‒E; Supplementary Figure S2B). Consistently, the p53LCs failed to suppress the colony formation compared with wt p53 (Figure 1F and G). This was further confirmed in H1299 and HCT116^p53–/–^ cells stably expressing Flag-p53-393*78 as measured by cell proliferation and colony formation assays (Supplementary Figure S2C‒H). Since overexpression of either of the two mutants in p53-free lung or colon cancer cells did not significantly enhance their proliferation and colony formation (Figure 1; Supplementary Figure S2), the p53LCs do not possess apparent GOF activity in the cancer cells tested. These results demonstrate that the p53LCs lose wt p53 functions, but without apparent GOF.

### p53LCs possess DN effects on wt p53 function

**Figure 2:**
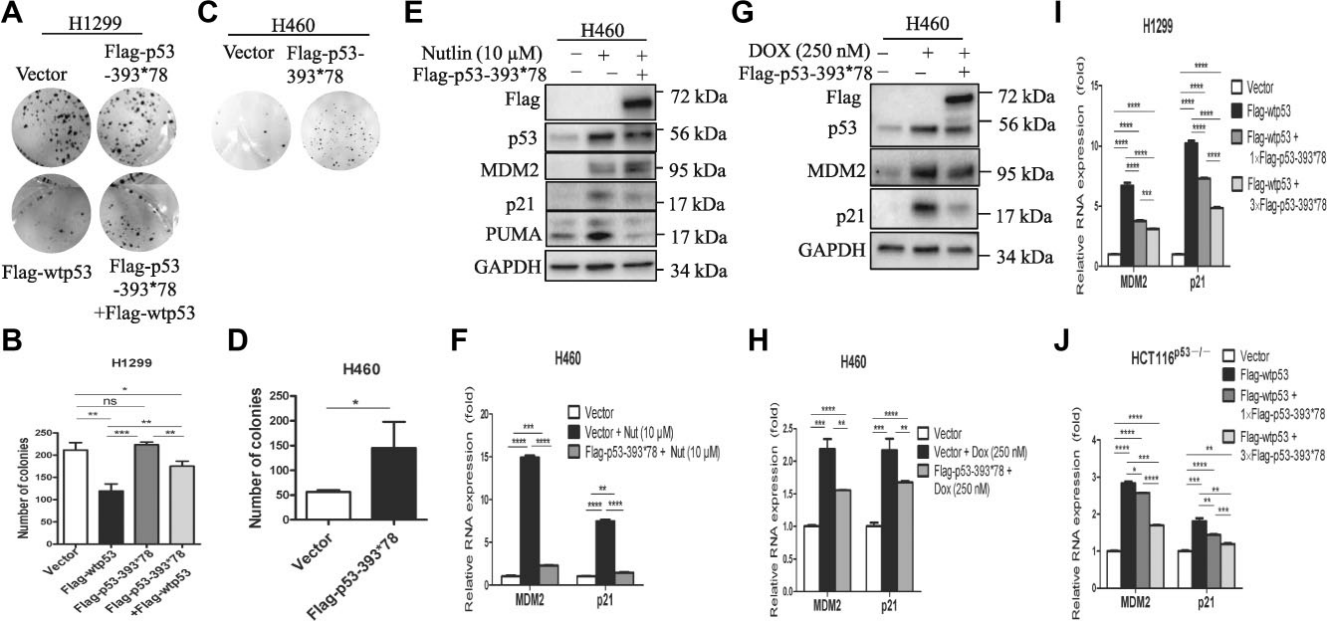
p53-393*78 impairs wt p53 functions. (**A‒D**) H1299 cells (**A** and **B**) or H460 cells (**C** and **D**) transfected with plasmids as indicated were seeded on six-well plates and cultured with 500 µg/ml G418 for 10–14 days. (**A** and **C**) Colonies were fixed for crystal violet staining. (**B** and **D**) The number of colonies was quantified. (**E‒H**) H460 cells transfected with Flag vector or Flag-p53-393*78 were treated with nutlin (Nut) (**E** and **F**) or doxorubicin (DOX) (**G** and **H**) for 16 h and then harvested for IB analysis with indicated antibodies (**E** and **G**) and RT–qPCR analysis (**F** and **H**), respectively. (**I** and **J**) H1299 (**I**) or HCT116^p53−/−^ (**J**) cells were transfected with wt p53 in the absence or presence of increasing amounts of p53-393*78. The mRNA levels of MDM2 and p21 were measured using RT–qPCR. Data are presented as mean ± SEM of triplicate experiments. *P < 0.05, **P < 0.01 by two-tailed *t*-test.

Next, we determined whether p53-393*78 and p53-374*48 might affect cancer cell survival and proliferation through DN effect by conducting a colony formation assay. As shown in Figure 2A and B, ectopic p53-393*78 alone in p53-free H1299 cells showed no effect on colony formation but attenuated the inhibitory effect of exogenous wt p53 on the colony formation of the cells. Also, it promoted colony formation in wt p53-containing H460 cells (Figure 2C and D). Similar DN effects were also observed for p53-374*48 in human p53-containing cancer cells and p53^–/–^ and MDM2^–/–^ double knockout mouse embryonic fibroblast (MEF) cells that expressed ectopic wt p53 (Supplementary Figure S3A‒D). Collectively, these data indicate that the p53LCs can exert their DN effects on the functions of their wt counterpart.

To determine whether the p53LCs could also exert their DN effects on endogenous wt p53, we transfected wt p53-containing H460 cells with Flag-p53-393*78 and then treated the cells with reagents to activate endogenous wt p53. As expected (Chao et al., 2016; Zhou et al., 2016), nutlin and doxorubicin drastically induced the protein level of p53 (Figure 2E and G) and the expression at both mRNA and protein levels of p53 target genes, MDM2, p21, and PUMA (Figure 2E‒H). Consistent with the results of Figure 2A‒D, the induction of these target genes was markedly mitigated in the presence of p53-393*78 (Figure 2E‒H) or p53-374*48 (Supplementary Figure S3E‒J). Also, p53-393*78 exerted a DN effect on the transcriptional activity of exogenous wt p53, as it suppressed Flag-wtp53-induced MDM2 and p21 mRNA levels in a dose-dependent manner (Figure 2I and J). These results demonstrate that the p53LCs can exert their DN effects on wt p53 function by suppressing the transcriptional activity of both exogenous and endogenous p53s even in response to stresses. This is in line with the fact that p53 mutations often occur heterozygously with one wt TP53 allele in different human cancers (Supplementary Figure S1), suggesting that the p53LCs could dominantly negate the function of wt p53 in the tumors.

### p53LCs predominantly reside and retain wt p53 in the cytoplasm

**Figure 3:**
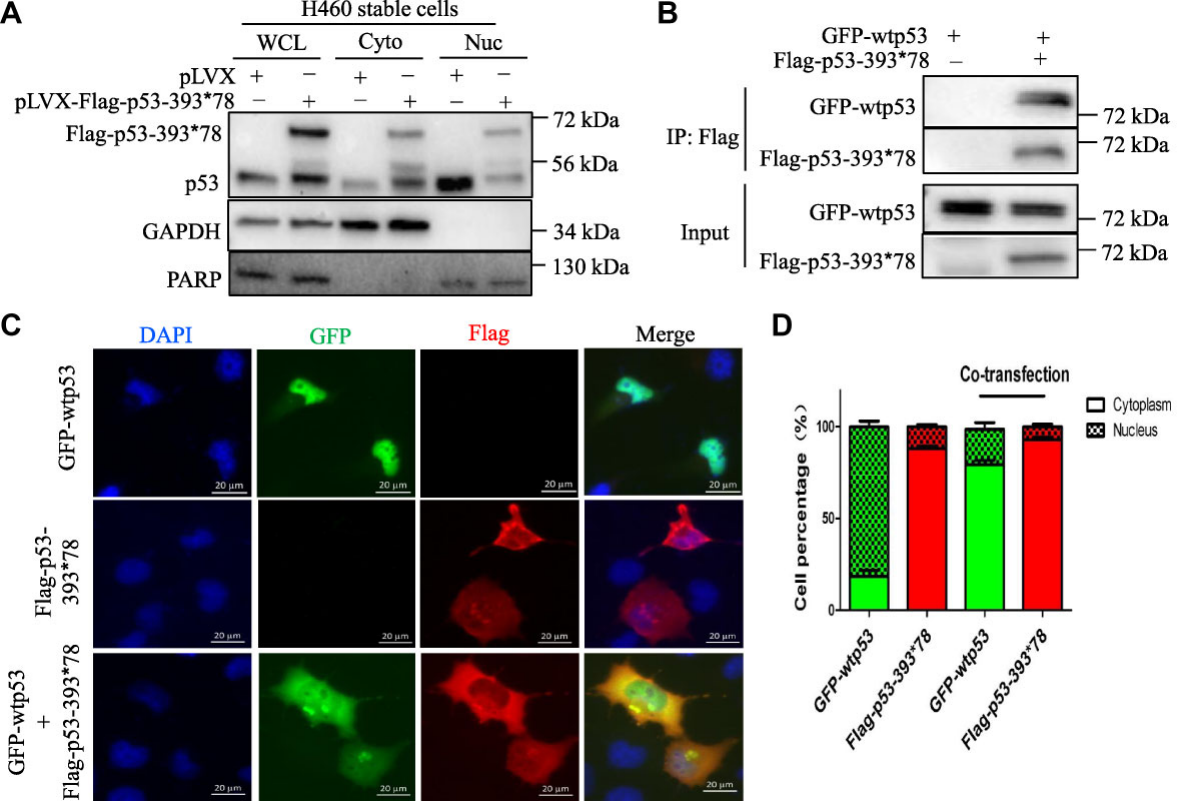
p53-393*78 is predominantly present in the cytoplasm and interacts with wt p53. (**A**) Nuclear and cytoplasmic distributions of ectopic wt p53 and p53-393*78. H460 cells transfected with either Flag-p53 or Flag-p53-393*78 were subjected to fractionation. Nuclear and cytoplasmic proteins were analyzed by IB assays with antibodies as indicated. GAPDH and PARP were used as the cytoplasmic and nuclear markers, respectively. (**B**) H1299 cells were transfected with GFP-wtp53 in the presence or absence of Flag-p53-393*78 followed by co-IP assays using anti-Flag antibodies and IB analysis with indicated antibodies. (**C** and **D**) H1299 cells were transfected with GFP-p53 and Flag-p53-393*78 alone or together for 36 h, and then subjected to IF staining with the anti-Flag antibody (red) and counterstained with DAPI. The representative images were shown in **C** and quantification data are shown in **D** (*n* ≥ 120).

Next, we wanted to determine how these p53LCs dominantly negate wt p53 activity in cells. Subcellular distribution is crucial for the control of p53 transcriptional activity, and nuclear transport is dependent on three NLSs located in the C-terminal region of p53 (Marchenko et al., 2010). To test whether the extended C-terminus of p53LCs might affect their nuclear localization, we performed fractionation assays using H460 cells. While the majority of endogenous wt p53s were detected in the nucleus, most of the p53-393*78 proteins were detected in the cytoplasm, by IB analysis of cellular fractions (Figure 3A). In the presence of ectopic p53-393*78, the nuclear wt p53 level was drastically reduced, while the cytoplasmic p53 level was increased correspondingly (Figure 3A). Consistent with this result, Flag-p53-393*78 (Figure 3B) or Flag-p53-374*48 (Supplementary Figure S4A) formed a complex with GFP-wtp53 as detected by immunoprecipitation (IP) assays. Their interactions and colocalization in the cytoplasm were further confirmed by immunofluorescence (IF) staining, as wt p53 was colocalized with either p53-393*78 or p53-374*48 predominantly in the cytoplasm, when they were coexpressed in the cells, whereas wt p53 alone was mostly detected in the nucleus (Figure 3C and D; Supplementary Figure S4B). These results demonstrate that p53-393*78 and p53-374*48 mostly reside in the cytoplasm and can retain wt p53 in the cytoplasm. These observations could also partially explain the LOF and DN effects of p53LCs on their wt counterpart.

### p53LCs prevent wt p53 from binding to its target promoters

**Figure 4:**
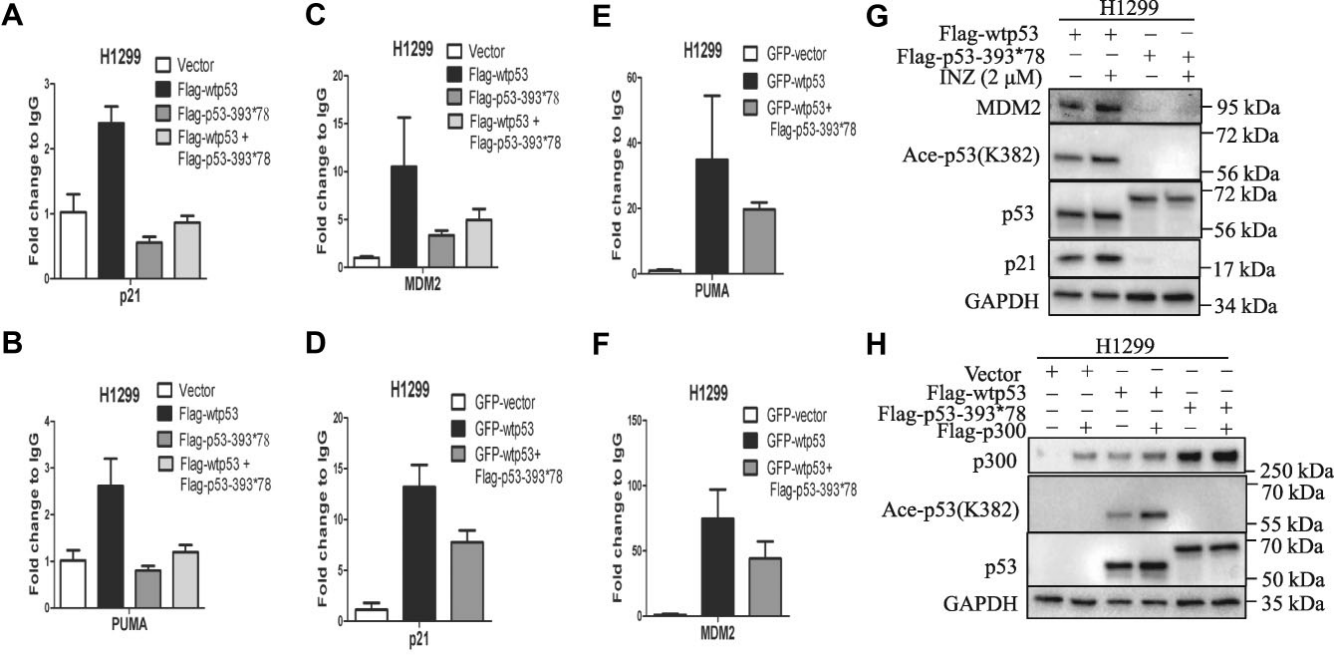
p53-393*78 loses DNA-binding activity, inhibits wt p53’s DNA binding, and is not acetylated in cells. (**A‒C**) H1299 cells were transfected with vector, Flag-wtp53, Flag-p53-393*78 alone, or the latter two together and harvested 48 h after transfection for ChIP assays with the anti-Flag antibody or control IgG followed by RT–qPCR analysis. (**D‒F**) H1299 cells were transfected with GFP-wtp53 in the absence or presence of Flag-p53-393*78 and harvested 48 h after transfections for ChIP assays using GFP antibody or control IgG followed by RT–qPCR analysis. (**G**) H1299 cells were transfected with Flag-wtp53 or Flag-p53-393*78 for 24 h and then treated with vehicle or inauhzin (INZ) for an additional 16 h. Cells were harvested for IB analysis with indicated antibodies. (**H**) H1299 cells were transfected with Flag-wtp53 or Flag-p53-393*78 with or without Flag-p300 and harvested 48 h after transfection for IB analysis with indicated antibodies.

Next, we determined whether the nuclear p53LCs could bind to the p53-responsive DNA elements and whether they could affect the ability of wt p53 to bind to the DNA elements in cells. To this end, we performed a set of chromatin immunoprecipitation (ChIP) assays in H1299 cells transfected with the Flag-empty vector, Flag-wtp53, Flag-p53-393*78 alone, or the latter two together. Interestingly, p53-393*78 was unable to bind to the promoters of the p53 target gene p21 and Puma, though weakly bound to the MDM2 promoter, compared with wt p53 (Figure 4A‒C), even though it has an intact DBD (Figure 1A). Remarkably, p53-393*78 suppressed the ability of wt p53 to bind to all of the target gene promoters tested here (Figure 4A‒C). This result was reproduced when GFP-wtp53 and Flag-p53-393*78 were used for the same experiment to avoid the possible Flag tag competition (Figure 4D‒F). These results demonstrate that the p53LC loses its ability to bind to p53-responsive DNA elements and also inhibits its wt counterpart's DNA-binding activity in cells, offering a second mechanism underlying the p53LCs’ LOF and DN effects on their wt counterpart.

### p53LCs cannot be acetylated at their C-terminus

Acetylation of the p53’s C-terminal lysine residues, such as K382, plays important roles in activating (Gu and Roeder, 1997; Feng et al., 2005) and stabilizing p53 (Kobet et al., 2000; Ito et al., 2001). To assess whether the extended C-terminus of p53LCs might affect their acetylation, we treated H1299 cells that expressed either Flag-wtp53 or Flag-p53-393*78 with a SIRT1 inhibitor inauhzin (INZ; Zhang et al., 2012) and then conducted IB analysis. As expected, the K382 acetylation and total p53 levels of Flag-wtp53 as well as the MDM2 and p21 levels were induced by INZ in the cells (Figure 4G). Strikingly, no acetylation was detected on Flag-p53-393*78 (Figure 4G) even though it contained all of the lysine residues (Figure 1A). However, surprisingly, p300 was co-immunoprecipitated with Flag-p53-393*78 or Flag-p53-374*48 as well as with wt p53 by the anti-p300 antibody in our co-IP‒IB assay (Supplementary Figure S4C). Even though p300 bound to all of the p53s, ectopic p300 only acetylated wt p53 but not the p53LCs (Figure 4H). This negative result for Flag-p53-393*78 acetylation was validated with the Pan anti-acetyl antibody that could recognize multiple C-terminal acetylated lysines of p53 (Gu and Roeder, 1997; Supplementary Figure S4D). Similarly, no acetylation on Flag-p53-374*48 by p300 was detected (Supplementary Figure S4D). Collectively, these results demonstrate that the C-terminal extension prevents p53LCs from being acetylated at least at their C-terminal lysines, which might account for another mechanism underlying LOF of these p53 mutants. This result also suggests that the extended C-terminus might cause the conformational alteration of the entire C-terminus of the p53 mutant, sheltering the target lysines from acetylation by p300, even though they bind to each other.

### p53LCs are not degraded by MDM2

The fact that p53LCs cannot be acetylated (Figure 4G and H; Supplementary Figure S4D) suggested that these mutant p53s might be less stable than wt p53, because nonacetylated p53s are more vulnerable for ubiquitination-mediated degradation by MDM2 (Kobet et al., 2000; Ito et al., 2001). To test the possibility, we first determined whether p53LCs could bind to MDM2 by performing a co-IP‒IB assay after transient transfection in H1299 cells. HA-MDM2 was co-immunoprecipitated with Flag-p53-393*78 or Flag-p53-374*48 by the anti-Flag antibody (Supplementary Figure S5A). Interestingly, more MDM2 molecules were pulled down with mutant p53s than with wt p53, even though their expression levels were equivalent as detected by straight IB analysis (Supplementary Figure S5A), suggesting that p53LCs might bind to MDM2 with a high affinity. However, the half-life of Flag-p53-393*78 or Flag-p53-374*48 surprisingly lasted longer than 2 h even in the presence of ectopic HA-MDM2 (Supplementary Figure S5B, C, F, and G), which was much longer than the half-life (∼30 min) of wt p53 (Chao et al., 2016). More surprisingly, p53-393*78 or p53-374*48 was still being ubiquitinated by MDM2 (Supplementary Figure S5D and H). This ubiquitination by MDM2 appeared to occur at K63 of ubiquitin for polyubiquitin chain formation in the p53LC, as the ubiquitination level of p53-393*78 was decreased when the His-Ub K63R, but not K48R, mutant was used (Supplementary Figure S5E). These results demonstrate that the p53LCs could interact with MDM2 and be ubiquitinated via K63, but not degraded by this E3 ubiquitin ligase.

### p53LCs desensitize wt p53-positive cancer cells to therapeutic agents

**Figure 5:**
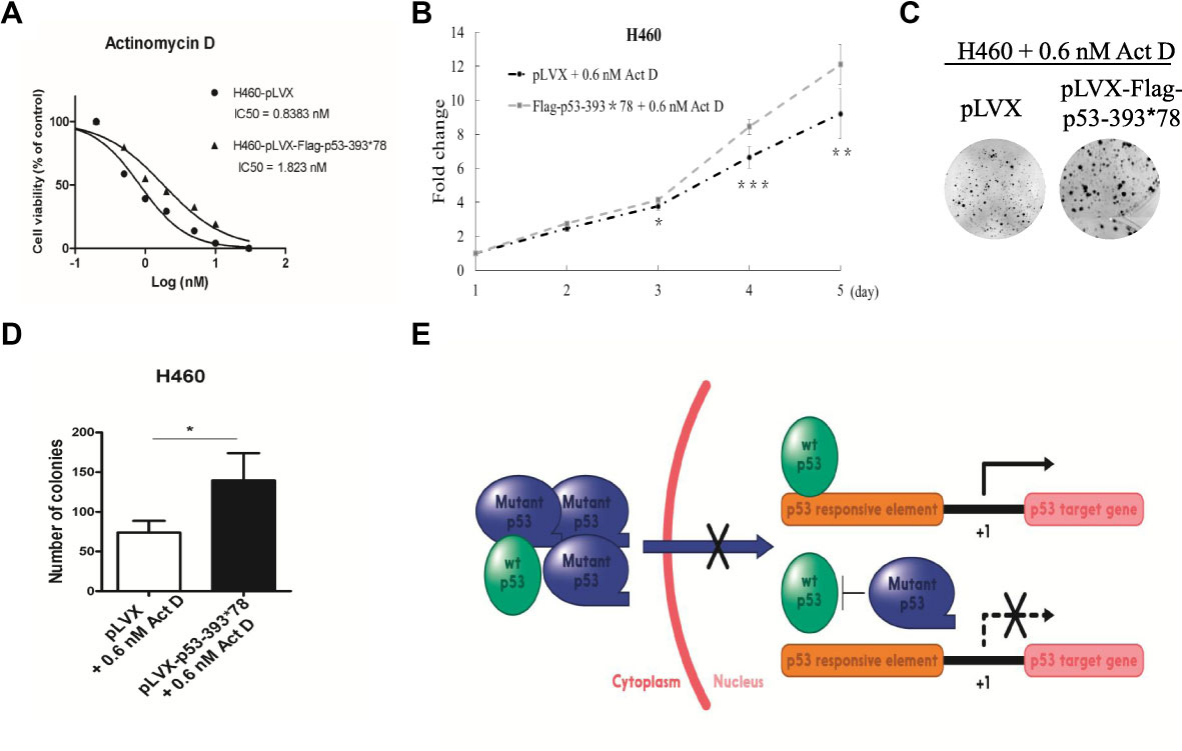
p53-393*78 desensitizes wt p53-bearing cancer cells to Act D treatment. (**A**) H460 cells stably expressing vector or Flag-p53-393*78 were treated with different concentrations of Act D for cell survival analysis. IC50 values are presented as mean ± SD (*n* = 6). (**B**) H460 cells stably expressing vector or Flag-p53-393*78 were seeded in 96-well plates and treated with 0.6 nM Act D. Cell viability was evaluated every 24 h by CCK8 assays. (**C** and **D**) H460 cells stably expressing vector or Flag-p53-393*78 were treated with 0.6 nM Act D for colony formation assays. (**C**) Colonies were fixed and stained with crystal violet solution. (**D**) The number of colonies was quantified. (**E**) A model depicting the DN effect of p53LCs on wt p53. A bar indicates ‘inhibition’, a cross presents ‘blocking’, while an arrow presents ‘activation’.

Since p53LCs possess LOF and DN effects as shown here, we wanted to determine whether p53LCs could confer chemoresistance to p53-activating drugs. To do so, we first tested the half-inhibitory concentration (IC50) of wt p53-containing H460 cells that stably expressed a vector or Flag-p53-393*78 in response to actinomycin D (Act D) treatment. Interestingly, the IC50 was significantly higher for the Flag-p53-393*78 cells than for control cells (Figure 5A). Next, we performed cell proliferation and colony formation assays using the same stable cell lines that were treated with 0.6 nM Act D. As shown in Figure 5B‒D, when challenged with Act D, the cells that expressed Flag-p53-393*78 exhibited significant advantage in cell proliferation or colony formation as compared with control cells. Consistently, when challenged with nutlin, H460 cells that expressed Flag-p53-74*48 exhibited significant advantage in cell proliferation and colony formation as compared with control cells (Supplementary Figure S6A‒C). These results, together with the results shown in Figure 2 and Supplementary Figure S3, demonstrate that the p53LCs can confer the resistance of wt p53-harboring lung cancer cells to the p53-activating agents through their DN effects.

## Discussion

Mutation of the tumor suppressor p53 is one of the most frequent events in human cancers. Here, we identified a novel frame-shift mutation at G374 of p53, yielding a longer peptide product than wt p53, from a prostate cancer sample (Figure 1A). Strikingly, our further analysis of publicly available data sets (Cerami et al., 2012; Gao et al., 2013) revealed 40 more cases of this type of p53 mutations with an extended C-terminus across a variety of human cancers, most of which are heterozygous with a wt TP53 allele in the same tumor (Supplementary Figure S1). Given the facts that p53’s C-terminus plays an important role in regulating p53 functions and little is known about this C-terminus-extended mutation, we studied two representative mutants, i.e. p53-374*48 and p53-393*78. Our results clearly showed that the p53LCs failed to transcriptionally activate p53 target genes, such as MDM2 and p21, or inhibit proliferation and survival of cancer cells (Figure 1; Supplementary Figure S2). Also, when wt p53-containing cancer cells were challenged with p53-activating drugs, ectopic p53LCs drastically mitigated the wt p53-dependent toxic effects of these drugs (Figures 2 and 5; Supplementary Figure S3). These results demonstrate that the p53LCs possess LOF and DN effects on their wt counterpart.

These findings are surprising and interesting, as these p53LCs still have the intact N-terminal TAD and central DBD. Normally, p53 functions as a tetramer in the nucleus to regulate the transcription of its target genes (Shaulsky et al., 1990; Joerger and Fersht, 2016). It has been shown that the C-terminal domain plays a critical role in the regulation of p53 tetramerization and nuclear localization (McCoy et al., 1997; Nakamura et al., 2000). Mutations at K372R, K373R, K381R, and K382R in the p53 C-terminus led to the cytoplasmic retention of the p53 protein (Nakamura et al., 2000). Similar to this 4KR mutant, the p53LCs also resided predominantly in the cytoplasm, whereas wt p53 was predominantly present in the nucleus (Figure 3A). This in part explains why these longer p53s lose wt p53’s transcriptional activity and also suggests that the extra C-terminus could cause a structurally conformational change that might prevent the recognition of the NLS peptide of p53 by the nuclear import machinery, though this remains to be investigated. We speculated that this conformational change might be independent of the aa sequence of the extended C-terminus of the p53LC, as the extra C-terminal aa sequences of the two p53LCs are distinct (Figure 1A). However, the extra C-terminus did not affect the ability of the p53LCs to form a complex with wt p53 (Figure 3B; Supplementary Figure S4A) likely via their tetramerization domains (Shaulsky et al., 1990; Joerger and Fersht, 2016). This ability accounts for their DN effects on wt p53, as the p53LCs retained a large portion of wt p53s in the cytoplasm (Figure 3B‒D; Supplementary Figure S4B). Therefore, these results suggest that the C-terminal extension with 48 or 78 different amino acids (probably even just 40 amino acids) (Figure 1A; Supplementary Figure S1) could cause a structural change that ‘shields’ the NLS site of p53 from recognition by the nuclear import machinery, but this change does not appear to affect p53 tetramerization (Figure 1A). Thus, p53LCs could dominantly negate the activity of wt p53 by retaining the latter in the cytoplasm (Figure 5E).

Consistent with their inability to activate the transcription of their target genes (Figure 2; Supplementary Figure S3), p53-393*78 as tested here also lost its ability to bind to the p21 and Puma promoters as well as partially to the MDM2 promoter as measured by ChIP assays (Figure 4A‒C). Again, p53-393*78 dominantly suppressed the binding of wt p53 to these target promoters (Figure 4A‒F). Acetylation of p53 plays important roles in regulating its stability and activity (Pfister and Prives, 2017; Liu et al., 2019). Deacetylase inhibitors and CBP/p300 acetyltransferase have been shown to increase p53 acetylation and sequence-specific DNA-binding activity (Gu and Roeder, 1997; Liu et al., 2019). Also, the CTD enables DNA binding in a sequence-dependent manner that is drastically altered by either its modification or its deletion (Laptenko et al., 2015). Therefore, we tested the effect of INZ, a SIRT1 inhibitor (McCoy et al., 1997), and ectopic p300 on acetylation of p53-393*78 or p53-374*48 in cells. Interestingly, both INZ and ectopic p300 failed to lead to acetylation of p53-393*78, though they did increase acetylation of wt p53 and activate its activity (Figure 4G and H). However, unexpectedly, neither p53-393*78 (Figure 4G and H) nor p53-374*48 (Supplementary Figure S4D) was acetylated at its C-terminus as detected with either anti-acetyl-K382 or Pan anti-acetyl antibodies, even though these p53LCs, particularly p53-393*78, harbor C-terminal lysine residues (Figure 1A) and were still able to bind to p300 (Supplementary Figure S4C). Since p53s bind to p300 via their N-terminal domains (Gu and Roeder, 1997) that are intact in the p53LCs, these results indicate that p53-393*78 and p53-374*48 are not accessible to acetylation-mediated activation and thus provide another mechanism underlying the LOF and DN phenotypes of p53LCs in cancer cells, though it remains to be found how exactly p53LCs fail to bind to their target promoters and how to be good substrates for p300.

Surprisingly, even though the p53LCs could still bind to and be ubiquitinated by MDM2 in cancer cells (Supplementary Figure S5A, D, and H), they were not degraded by this E3 ubiquitin ligase, as their half-lives were not reduced even in the presence of ectopic MDM2 (Supplementary Figure S5B, C, F, and G). This can be partially explained by the fact that MDM2-specific ubiquitination of p53 does not necessarily lead to degradation of p53, pending on the chain length and chain linkage. We then tested whether K63 of His-Ub is crucial for the ubiquitination of p53-393*78. Indeed, K63 of His-Ub appeared to be utilized for p53LC ubiquitination by MDM2 (Supplementary Figure S5E), which does not lead to degradation (Hock and Vousden, 2014). Taken together, these results demonstrate that p53LCs are resistant to MDM2-mediated degradation, though it remains to be studied what would be the detailed underlying mechanisms and which lysine residues within the mutant p53s are ubiquitinated.

Although the p53LCs display no GOF in p53-null lung and colon cancer cells (Figures 1 and 2; Supplementary Figure S2), their DN effects on wt p53 activity have clinical implications. The p53LCs often coexist with a wt TP53 allele in various cancers (Supplementary Figure S1). The p53LCs could potentially cause the resistance of these cancers to chemotherapy by dominantly suppressing the activity of wt p53. Indeed, overexpression of the p53LCs not only suppressed the transcriptional activity of exogenous and endogenous wt p53 even in response to cisplatin, nutlin-3, or DOX (Figure 2; Supplementary Figure S3), but also showed significant survival advantage as evidenced by increasing the IC50 of the cancer cells treated with either act D or nultin-3 (Figure 5A‒D; Supplementary Figure S6A‒C). Although our study is limited with overexpression of these mutant p53s in cancer cells mainly due to the fact that there is no such cancer cell line that harbors available p53LC, our findings that p53LCs can act as the DN suppressor of wt p53 (Figure 5E) are valuable to clinical therapeutic designs for those cancers that harbor these p53 mutations and also offer new targets for future development of personalized anticancer drugs against these cancers. Such mutations, when present in the germline, may represent a novel cause of the Li‒Fraumeni syndrome.

## Materials and methods

### Cloning p53-374*48 and p53-393*78

We first synthesized the p53-374*48 and p53-393*78 genes by working with Origene Technologies and then subcloned them into their mammalian expression vectors pcDNA3-2xFlag and pLVX, respectively (Figure 1A; Supplementary Figure S2A). Their complementary DNA (cDNA) sequences were confirmed by DNA sequencing, which showed no point mutation in other domains of the two proteins (see Supplementary Sequence data). We used the synthetic genes, mainly because there were not any cancer cells that harbor this type of p53 mutations available, and thus cloning them out from cancer cells by a reverse genetic approach was not feasible.

### Cell culture and transient transfection

H460 (wt p53) and H1299 (p53-null) cells were purchased from American Type Culture Collection. HCT116^p53+/+^ and HCT116^p53^^−^^/^^−^ cells were generous gifts from Dr Bert Vogelstein at John Hopkins University School of Medicine. MEF^p53^^−^^/^^−^^;Mdm2^^−^^/^^−^ cells were generous gifts from Dr Guillermina Lozano from the University of Texas MD Anderson Cancer Center. STR profiling was performed to ensure cell identity. No mycoplasma contamination was found. All cells were cultured in Dulbecco's modified Eagle's medium (DMEM) supplemented with 10% fetal bovine serum (Gibco), 1% penicillin and streptomycin (Gibco). All cells were maintained at 37°C in a 5% CO_2_ humidified atmosphere. Cells seeded on the plate overnight were transfected with plasmids as indicated using TurboFect transfection reagent following the manufacturer's protocol (Thermo Scientific). Cells were harvested at 30–48 h posttransfection for future experiments.

### ChIP

ChIP assay was performed using antibodies as indicated (Liao and Lu, 2013). Briefly, 5 × 10^7^ H1299 cells were seeded in a 15-cm culture dish, transfected with p53LC plasmid for 48 h, and then cross-linked with 1% formaldehyde (37%) at 37°C for 10 min. Cells were pelleted by centrifugation at 1000× *g* for 5 min and sonicated to shear DNA to the length of 500‒1000 bp. Sonicated cell lysates were centrifuged at 16000× *g* for 5 min and the supernatants after being precleaned with protein A beads and single-stranded DNA were incubated overnight at 4°C with 2‒5 µg antibody against Flag (Sigma-Aldrich, F1804)/GFP (Santa Cruz Biotechnology, sc-9996, B-2) or normal mouse immunoglobulin G (IgG) in the presence of protein A/G Sepharose beads (Invitrogen). Precipitated chromatin was eluted with 400 µl elution buffer (1% sodium dodecyl sulphate [SDS], 0.1 M NaHCO3), incubated at 65°C for 4 h or overnight in the presence of 10 µg RNase A and 200 mM NaCl, and used for DNA extraction with miniprep DNA Spin Kit. RT–qPCR was performed using an SYBR Green qPCR Kit (Bio-Rad). The following primer sets were used: p21, Fw-5′-GCTCCCTCATGGGCAAACTCACT-3′ and Rv-5′-TGGCTGGTCTACCTGGCTCCTCT-3′; PUMA, Fw-5′-CTGTGGCCTTGTGTCTGTGAG-3′ and Rv-5′-CTAGCCCAAGGCAAGGAGGAC-3′; and MDM2, Fw-5′-GGTTGACTCAGCTTTTCCTCTTG-3′ and Rv-5′-GGAAAATGCATGGTTTAAATAGCC-3′.

### RT–qPCR analysis

Total RNA was isolated from cells using Trizol (Invitrogen) following the manufacturer's protocol. Total RNAs of 0.5‒1 µg were used as templates for reverse transcription using Anchored Oligo(dT)_20_ primers and M-MLV reverse transcriptase (Promega). RT–qPCR was conducted using SYBR Green Mix according to the manufacturer's protocol (BioRad). The primers for cDNA detection are as follows: GAPDH, 5′-CAGGGCTGCTTTTAACTCTGGT-3′ and 5′-GATTTTGGAGGGATCTCGCT-3′; p21, 5′-TGTCCGTCAGAACCCATGC-3′ and 5′-AAAGTCGAAGTTCCATCGCTC-3′; PUMA, 5′-ACAGTACGAGCGGCGGAGACAA-3′ and 5’-GGCGGGTGCAGGCACCTAATT-3′; and MDM2, 5′-ATGAATCCCCCCCTTCCAT-3′ and 5′-CAGGAAGCCAATTCTCACGAA-3′.

### Cell viability assay

To assess the long-term cell survival, the Cell Counting Kit-8 (CCK-8) (Dojindo Molecular Technologies) was used according to the manufacturer's instructions. Cell suspensions were seeded at 2000 cells per well in 96-well culture plates at 12 h posttransfection. Cell viability was determined by adding CCK-8 at a final concentration of 10% to each well, and the absorbance of the samples was measured at 450 nm using a Microplate Reader (Molecular Device, SpecrtraMax M5e) every 24 h as one point and measured for 5 days. The IC50 values were calculated by Prism 5 (GraphPad Software).

### Colony formation assay

Cancer cells (50% confluence) were transfected with specific or vector plasmid for 12–18 h. After trypsinizing cells, the same number of cells was seeded on each 6-well plate. Media added with 500 µg/ml G418 were changed every 3 days until colonies were visible. Cells were then fixed with methanol and stained with crystal violet solution at room temperature for 30 min. ImageJ (NIH) was used for quantification of the colonies.

### IB analysis

Cells were harvested and lysed in lysis buffer consisting of 50 mM Tris/HCl (pH7.5), 0.5% Nonidet P-40, 1 mM ethylenediaminetetraacetic acid, 150 mM NaCl, 1 mM dithiothreitol (DTT), 0.2 mM phenylmethylsulfonyl fluoride (PMSF), and a protease inhibitor cocktail. Equal amounts of clear cell lysate (20–80 µg) were used for IB analyses. Each IB experiment was performed using the same blot, unless otherwise indicated.

### IP assay

IP was conducted using antibodies as indicated. Briefly, ∼500–1000 µg of protein was incubated with the indicated antibody at 4°C for 4 h or overnight. Protein A or G beads (Santa Cruz Biotechnology) were then added, and the mixture was incubated at 4°C for additional 1‒2 h. Beads were washed at least three times with lysis buffer. Bound proteins were detected by IB with antibodies.

### IF staining

Cells were fixed in 4% paraformaldehyde for 20 min, followed by permeabilization in 0.5% Triton X-100 for 20 min. The fixed cells were blocked with 8% bovine serum albumin for 30 min, and then the cells were incubated with indicated antibodies (Flag, 1:1000 dilution) at 4°C overnight. Cells were then washed and incubated with the corresponding secondary antibody and 4′-6-diamidino-2-phenylindole (DAPI) for nuclear staining. The cellular localization of wt p53 or mutant p53LCs was examined under a fluorescence microscope (ZEISS, Axiovert 200M).

### Cell fractionation

This experiment was performed as described previously (Liao et al., 2014). Briefly, ∼10^6^ cells were collected, washed twice with phosphate buffered saline, and resuspended in 1 ml buffer A (10 mM HEPES–KOH, pH 7.9, 1.5 mM MgCl_2_, 10 mM KCl, and 0.5 mM DTT) for 30 min on ice. PMSF was added to a final concentration of 0.2 mM, and the mixture was then Dounce homogenized until all cytoplasmic membranes were disrupted. For cytosolic isolation, cells were centrifuged at 228× *g* for 5 min at 4°C to obtain the supernatant as cytoplasm, and pellets were washed by Buffer A twice and stored as nuclear extracts.

### In vivo ubiquitination assay

H1299 cells were transfected with plasmids encoding HA-MDM2, His-Ub, wt p53, or p53LCs as indicated. At 48 h after transfection, cells were harvested and split into two aliquots, one for IB and the other for ubiquitination assay. Briefly, cell pellets were lysed in buffer I (6 M guanidinium‒HCl, 0.1 M Na_2_HPO_4_/NaH_2_PO_4_, 10 mM Tris–HCl, pH 8.0, and 10 mM β-mercaptoethanol) and incubated with Ni-NTA beads (QIAGEN) at room temperature for 4 h. Beads were washed once with buffer I, buffer II (8 M urea, 0.1 M Na_2_HPO_4_/NaH_2_PO_4_, 10 mM Tris‒HCl, pH 8.0, and 10 mM β-mercaptoethanol), and buffer III (8 M urea, 0.1 M Na_2_HPO_4_/NaH_2_PO_4_, 10 mM Tris‒HCl, pH 6.3, and 10 mM β-mercaptoethanol). Proteins were eluted from beads in buffer IV (200 mM imidazole, 0.15 M Tris‒HCl, pH 6.7, 30% glycerol, 0.72 M β-mercaptoethanol, and 5% SDS). Eluted proteins were analyzed by IB with the indicated antibodies.

### Generation of stable cell lines

Lentiviral plasmids based on pLVX were packaged with the third-generation packaging system. Briefly, pLVX plasmids without or with p53LC2, along with the packaging plasmids pMDLg/Prre, pRSV-Rev, and pMD2.G, were transfected into 293T cells. Cells were maintained at 37°C in a 5% CO_2_ humidified atmosphere for 72 h, and the supernatant was harvested and used for infecting H460 or H1299 cells. Infected cell plates were washed and changed with fresh media after overnight infection, and afterward changed with fresh media with puromycin (3 µg/ml) every 3 days till the forming of cell colonies. Then, colonies were separately picked for expansion culture.

### Statistics

All *in vitro* experiments were performed in biological triplicate. Student's two-tailed *t*-test was used to determine the mean difference among groups. *P* < 0.05 was considered statistically significant. Data are presented as mean ± SEM. All graphs and statistical calculations were done using GraphPad Prism software and Microsoft Excel.

## Supplementary Material

mjab078_Supplemental_File

## Notes

**Author contributions:** S.H., S.X.Z., and H.L. designed experiments and composed the manuscript; S.H. and B.C. performed experiments and acquired the data; J.W. and Y.Z. contributed to the animal study and protocol; O.S. and E.L. provided critical information on p53 mutations in prostate cancers; S.H., B.C., and S.X.Z. contributed to methodology development; Y.X. partly mentored S.H. The project was primarily operated by S.H. under supervision of S.X.Z. and H.L.; B.C., O.S., and S.X.Z. contributed to editing and proofreading of the manuscript; S.H. and H.L. drafted and revised the manuscript.
