# Supplementary material for: Cancer-derived C-terminus-extended p53 mutation confers dominant-negative effect on its wild-type counterpart
Source: J Mol Cell Biol. 2021 Dec 16;14(1):mjab078. doi: 10.1093/jmcb/mjab078 (PMC8964174; doi:10.1093/jmcb/mjab078)
Supplement: mjab078_Supplemental_File [file mjab078_supplemental_file.pdf]

Supplementary Information.

Supplementary Sequence Data

1. **p53-374*48 (p53LC1):**

Atggaggagccgcagtcagatcctagcgtcgagccccctctgagtcaggaaacattttcagacctatggaaactact
tcctgaaaacaacgttctgtcccccttgccgtcccaagcaatggatgatttgatgctgtccccggacgatattgaacaat
ggttcactgaagacccaggtccagatgaagctcccagaatgccagaggctgctccccccgtggcccctgcaccagc
agctcctacaccggcggcccctgcaccagccccctcctggcccctgtcatcttctgtcccttcccagaaaacctacca
gggcagctacggtttccgtctgggcttcttgcattctgggacagccaagtctgtgacttgcacgtactcccctgccctca
acaagatgttttgccaactggccaagacctgccctgtgcagctgtgggttgattccacacccccgcccggcacccgc
gtccgcgccatggccatctacaagcagtcacagcacatgacggaggttgtgaggcgctgcccccaccatgagcgct
gctcagatagcgatggtctggcccctcctcagcatcttatccgagtggaaggaaatttgcgtgtggagtatttggatga
cagaaacacttttcgacatagtgtggtggtgccctatgagccgcctgaggttggctctgactgtaccaccatccactac
aactacatgtgtaacagttcctgcatgggcggcatgaaccggaggcccatcctcaccatcatcacactggaagactcc
agtggtaatctactgggacggaacagctttgaggtgcgtgtttgtgcctgtcctgggagagaccggcgcacagagga
agagaatctccgcaagaaaggggagcctcaccacgagctgcccccagggagcactaagcgagcactgcccaaca
acaccagctcctctccccagccaaagaagaaaccactggatggagaatatttcacccttcagatccgtgggcgtgag
cgcttcgagatgttccgagagctgaatgaggccttggaactcaaggatgcccaggctgggaaggagccagggggg
agcagggctcactccagccacctgaagtccaaaaaggtcagtctacctcccgccataaaaaactcatgttcaagaca
gaagggcctgactcagactgacattctccacttcttgttccccactgacagcctcccacccccatctctccctcccctgc
cattttgggttttgggtctttga

2. **p53-393*78 (p53LC2):**

Atggaggagccgcagtcagatcctagcgtcgagccccctctgagtcaggaaacattttcagacctatggaaactact
tcctgaaaacaacgttctgtcccccttgccgtcccaagcaatggatgatttgatgctgtccccggacgatattgaacaat
ggttcactgaagacccaggtccagatgaagctcccagaatgccagaggctgctccccccgtggcccctgcaccagc
agctcctacaccggcggcccctgcaccagccccctcctggcccctgtcatcttctgtcccttcccagaaaacctacca
gggcagctacggtttccgtctgggcttcttgcattctgggacagccaagtctgtgacttgcacgtactcccctgccctca
acaagatgttttgccaactggccaagacctgccctgtgcagctgtgggttgattccacacccccgcccggcacccgc
gtccgcgccatggccatctacaagcagtcacagcacatgacggaggttgtgaggcgctgcccccaccatgagcgct
gctcagatagcgatggtctggcccctcctcagcatcttatccgagtggaaggaaatttgcgtgtggagtatttggatga
cagaaacacttttcgacatagtgtggtggtgccctatgagccgcctgaggttggctctgactgtaccaccatccactac
aactacatgtgtaacagttcctgcatgggcggcatgaaccggaggcccatcctcaccatcatcacactggaagactcc
agtggtaatctactgggacggaacagctttgaggtgcgtgtttgtgcctgtcctgggagagaccggcgcacagagga
agagaatctccgcaagaaaggggagcctcaccacgagctgcccccagggagcactaagcgagcactgcccaaca
acaccagctcctctccccagccaaagaagaaaccactggatggagaatatttcacccttcagatccgtgggcgtgag
cgcttcgagatgttccgagagctgaatgaggccttggaactcaaggatgcccaggctgggaaggagccagggggg
agcagggctcactccagccacctgaagtccaaaaagggtcagtctacctcccgccataaaaaactcatgttcaagac
agaagggcctgactcaggactgacattctccacttcttgttccccactgacagcctcccacccccatctctccctcccct
gccattttgggttttgggtctttgaacccttgcttgcaataggtgtgcgtcagaagcacccaggacttccatttgctttgtc
ccggggctccactgaacaagttggcctgcactggtgttttgttgtggggaggaggatggggagtaggacataccagc
ttagattttaa

Supplementary Figure 1.

A

p53 C-terminus extension mutants in cBioportal databases.

Study	Sample ID	Cancer Type	Protein Change	Zygosity
Colorectal Adenocarcinoma (DFCI, Cell Reports 2016)	coadread_dfci_2016_523	Colorectal Adenocarcinoma	K373Rfs*49	
MSK-IMPACT Clinical Sequencing Cohort (MSKCC, Nat Med 2017)	P-0010420-T01-IM5	Cutaneous Squamous Cell Carcinoma	K373Rfs*49	Heterozygous
Breast Cancer (METABRIC, Nature 2012 & Nat Commun 2016)	MB-5518	Breast Invasive Ductal Carcinoma	G374Afs*43	
			G374Vfs*48	
MSK-IMPACT Clinical Sequencing Cohort (MSKCC, Nat Med 2017)	P-0005615-T02-IM5	Glioblastoma Multiforme	S376Pfs*45	Heterozygous
TMB and Immunotherapy (MSKCC, Nat Genet 2019)	P-0015785-T01-IM6	Lung Adenocarcinoma	R379Sfs*39	
TMB and Immunotherapy (MSKCC, Nat Genet 2019)	P-0008718-T01-IM5	Bladder Urothelial Carcinoma	H380Ifs*42	Heterozygous
Metastatic Colorectal Cancer (MSKCC, Cancer Cell 2018)	P-0007669-T01-IM5	Colorectal Adenocarcinoma	H380Ifs*42	Heterozygous
MSK-IMPACT Clinical Sequencing Cohort (MSKCC, Nat Med 2017)	P-0007651-T01-IM5	Leiomyosarcoma	H380Qfs*42	Heterozygous
Bladder Urothelial Carcinoma (TCGA, PanCancer Atlas)	TCGA-GC-A3RC-01	Bladder Urothelial Carcinoma	K382Nfs*40	
Breast Cancer (MSK, Cancer Cell 2018)	P-0015630-T01-IM6	Breast	K382Nfs*40	
Colorectal Adenocarcinoma (DFCI, Cell Reports 2016)	coadread_dfci_2016_2944	Colorectal Adenocarcinoma	K382Nfs*40	
Colorectal Adenocarcinoma (DFCI, Cell Reports 2016)	coadread_dfci_2016_306	Colorectal Adenocarcinoma	K382Nfs*40	
Metastatic Colorectal Cancer (MSKCC, Cancer Cell 2018)	P-0003999-T01-IM5	Colorectal Adenocarcinoma	K382Nfs*40	Heterozygous
Metastatic Colorectal Cancer (MSKCC, Cancer Cell 2018)	P-0012313-T01-IM5	Colorectal Adenocarcinoma	K382Nfs*40	Heterozygous
Cholangiocarcinoma (MSK, Clin Cancer Res 2018)	P-0008025-T01-IM5	Extrahepatic Cholangiocarcinoma	K382Nfs*40	Heterozygous
MSK-IMPACT Clinical Sequencing Cohort (MSKCC, Nat Med 2017)	P-0004512-T01-IM5	Leiomyosarcoma	K382Nfs*40	Heterozygous
Non-Small Cell Lung Cancer (MSK, JCO 2018)	P-0002794-T02-IM5	Lung Adenocarcinoma	K382Nfs*40	Heterozygous
Brain Lower Grade Glioma (TCGA, Provisional)	TCGA-DU-8167-01	Oligoastrocytoma	K382Nfs*40	
Metastatic Prostate Cancer (SU2C/PCF Dream Team, Cell 2015)	MO_1124	Prostate Adenocarcinoma	K382Nfs*40	Heterozygous
MSK-IMPACT Clinical Sequencing Cohort in Prostate Cancer (MSK, JCO Precision Oncology 2017)	P-0003665-T01-IM5	Prostate Adenocarcinoma	K382Nfs*40	Heterozygous
MSK-IMPACT Clinical Sequencing Cohort (MSKCC, Nat Med 2017)	P-0006432-T01-IM5	Sarcomatoid Carcinoma of the Lung	K382Nfs*40	Heterozygous
Uterine Corpus Endometrial Carcinoma (TCGA, PanCancer Atlas)	TCGA-AP-A0LE-01	Uterine Endometrioid Carcinoma	K382Nfs*40	
Uterine Corpus Endometrial Carcinoma (TCGA, PanCancer Atlas)	TCGA-AX-A2HG-01	Uterine Endometrioid Carcinoma	K382Nfs*40	
Endometrial Cancer (MSK, 2018)	P-0011262-T01-IM5	Uterine Mixed Endometrial Carcinoma	K382Nfs*40	Heterozygous
Uterine Corpus Endometrial Carcinoma (TCGA, PanCancer Atlas)	TCGA-DI-A1BU-01	Uterine Mixed Endometrial Carcinoma	K382Nfs*40	
Head and Neck Squamous Cell Carcinoma (TCGA, Provisional)	TCGA-CQ-A4CD-01	Head and Neck Squamous Cell Carcinoma	L383Cfs*38	
Breast Cancer (METABRIC, Nature 2012 & Nat Commun 2016)	MB-4862	Breast Invasive Ductal Carcinoma	P390Lfs*32	
Pancreatic Adenocarcinoma (QCMG, Nature 2016)	ICGC_0277	Pancreatic Adenocarcinoma	P390Lfs*32	
Metastatic Colorectal Cancer (MSKCC, Cancer Cell 2018)	P-0010506-T01-IM5	Colorectal Adenocarcinoma	P390Lfs*79	Heterozygous
Ampullary Carcinoma (Baylor College of Medicine, Cell Reports 2016)	CAC_760	Ampullary Carcinoma	P390Rfs*80	
TCGA data for Esophagus-Stomach Cancers (TCGA, Nature 2017)	TCGA-IG-A97I-01	Esophageal Squamous Cell Carcinoma	D391Tfs*31	
Poorly-Differentiated and Anaplastic Thyroid Cancers (MSKCC, JCI 2016)	s_JJ_thy_019_P	Poorly Differentiated Thyroid Carcinoma	D391Tfs*31	
Stomach Adenocarcinoma (TCGA, PanCancer Atlas)	TCGA-VQ-A8E0-01	Intestinal Type Stomach Adenocarcinoma	S392Tfs*76	
MSK-IMPACT Clinical Sequencing Cohort in Prostate Cancer (MSK, JCO Precision Oncology 2017)	P-0004597-T02-IM5	Prostate Adenocarcinoma	D393Gfs*78	Heterozygous
MSK-IMPACT Clinical Sequencing Cohort (MSKCC, Nat Med 2017)	P-0002287-T01-IM3	Cancer of Unknown Primary	D393Rfs*78	Heterozygous
Diffuse Large B-Cell Lymphoma (Duke, Cell 2017)	DLBCL_DUKE_2839	Diffuse Large B-Cell Lymphoma, NOS	D393Rfs*78	
Endometrial Cancer (MSK, 2018)	P-0001460-T01-IM3	Uterine Serous Carcinoma/Uterine Papillary Serous Carcinoma	D393Rfs*78	Heterozygous
Breast Cancer (METABRIC, Nature 2012 & Nat Commun 2016)	MB-4644	Breast Invasive Ductal Carcinoma	*394Tfs*76	
MSK-IMPACT Clinical Sequencing Cohort (MSKCC, Nat Med 2017)	P-0006953-T01-IM5	Pancreatic Adenocarcinoma	*394Tfs*76	Heterozygous

List of p53 mutants with longer C-termini identified from cBioportal databases with the information, including datasets (Study), sample ID, Cancer type, Protein Change and Zygosity.

Supplementary Figure 2 p53LCs are loss-of-function mutations.

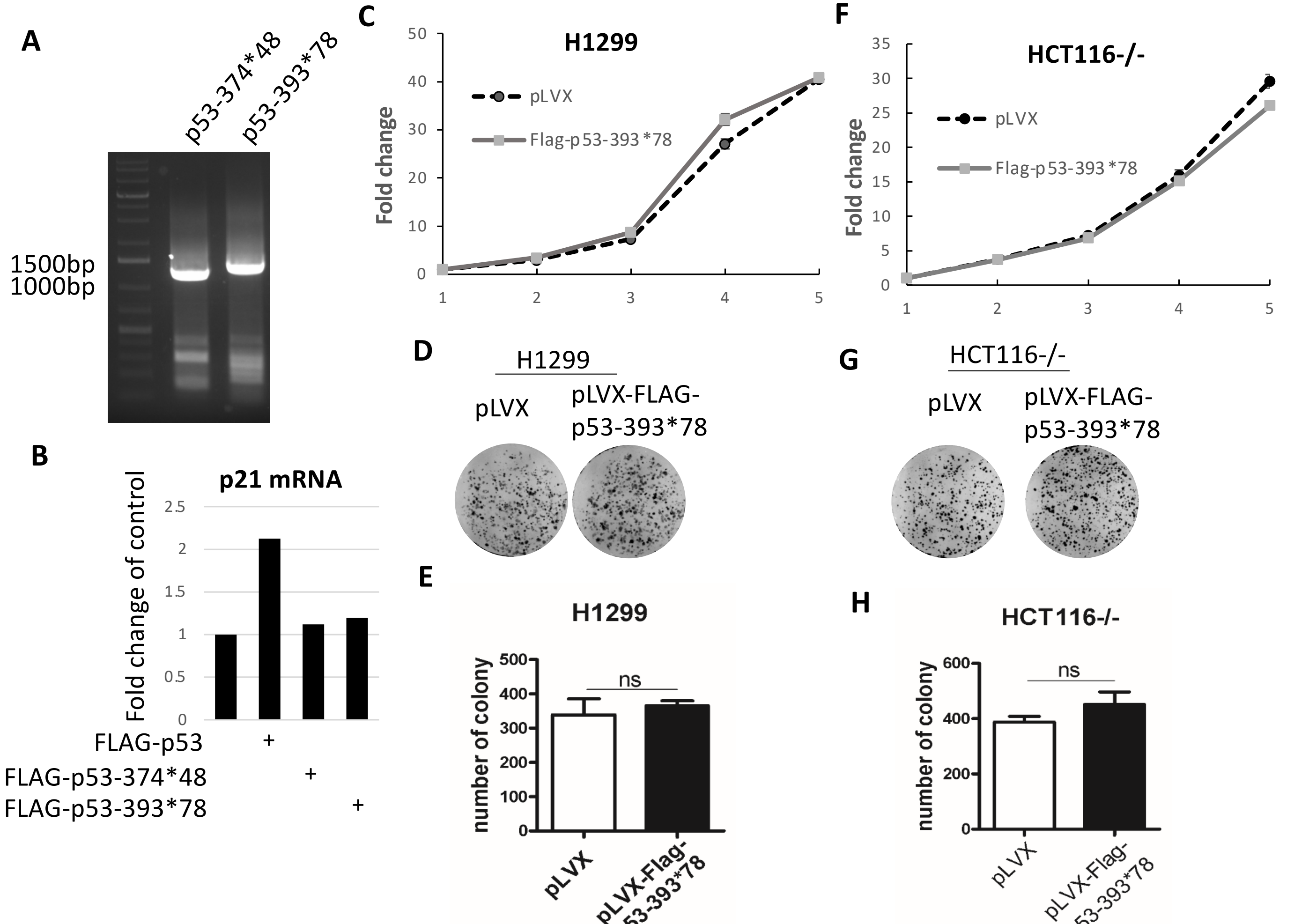

(A) An agarose gel stained with ethidium bromide confirms two mutant p53LC clones in different sizes, which were used for the experiments in this manuscript after verification of their DNA sequences. (B) H199 cells were transfected with Flag-p53, Flat-p53-3974*48 or Flat-p53-393*78 plasmids and harvested 48 h after transfection for RT-qPCR analysis. (C) H1299 cells that stably expressed vector or p53-393*78 were seeded in 96-well plate and cell viability was evaluated every 24 h by CCK-8 assays (mean ± SEM, n = 6). (D, E) H1299 cells stably expressing p53-393*78 were seeded on 6 well plates for 10–14 days, and colonies were stained with crystal violet solution. The number of colonies was quantified and shown in a graph (E). (F) HCT116p53-/- cells that stably expressed vector or p53-393*78 were seeded in 96-well plate and cell viability was evaluated every 24 h by CCK-8 assays (mean ± SEM, n = 6). (G, H) HCT116p53-/- cells that stably expressed vector or p53-393*78 were seeded on 6 well plates for 10–14 days, and colonies were stained with crystal violet solution and quantified as shown in a graph in panel H.

Supplementary Figure 3 p53-374*48 impairs WT-p53 function.

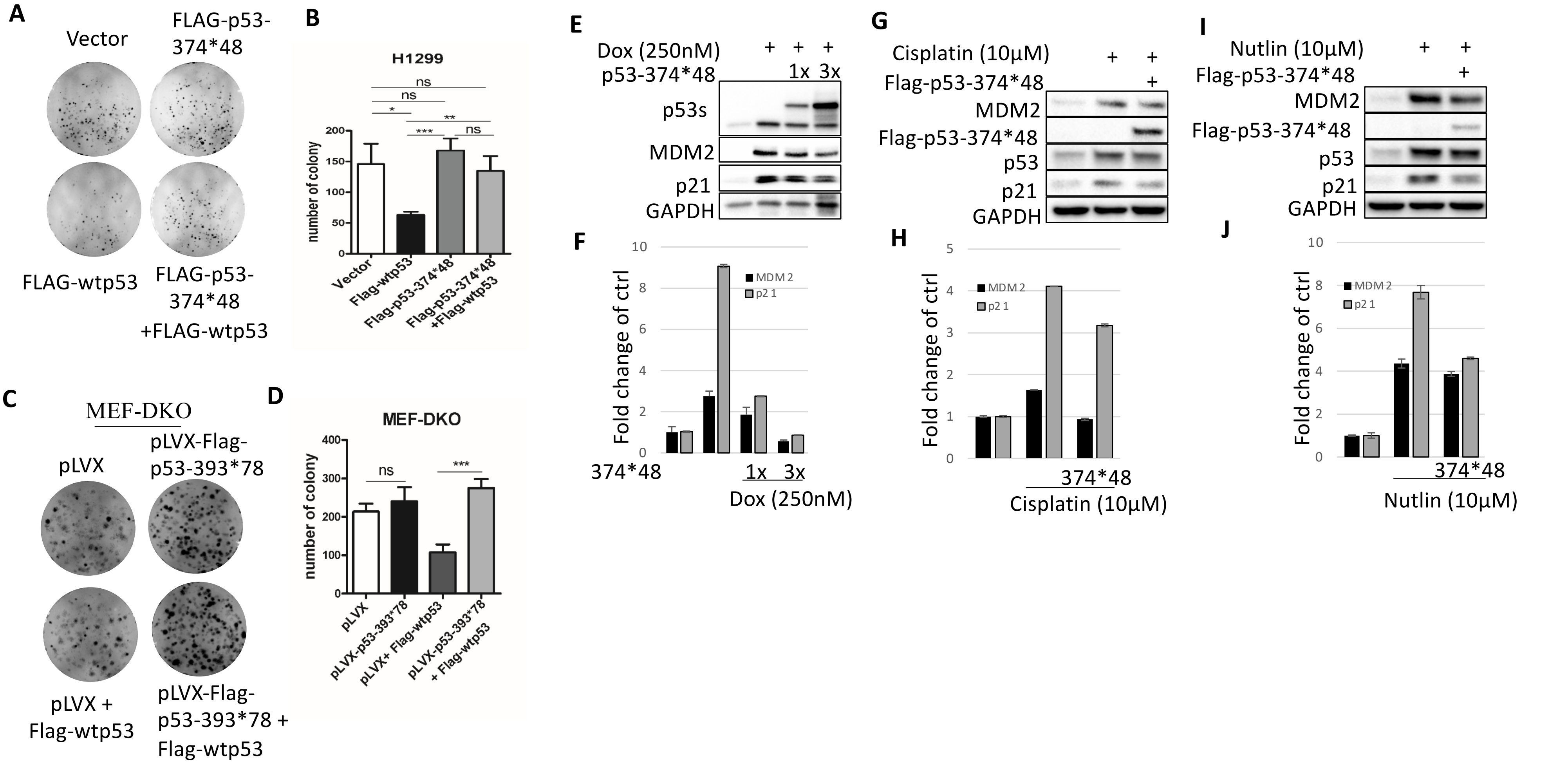

(A, B) H1299 cells transfected with wtp53 or p53-374*48 alone or in combination were seeded on 6 well plates and cultured with 500 μ g/ml G418 for 10–14 days, and colonies were stained with crystal violet solution and quantified as shown in panel B. (C, D) MEF(p53-/-, MDM2-/-) cells that stably expressed Flag-vector or Flag-p53-393*78 were transfected with wtp53 and then seeded on 6 well plates and cultured with 500 μ g/ml G418 for 10–14 days. Colonies were stained with crystal violet solution and quantified as shown in panel D. Data were presented as mean ± SEM of triplicate experiments.*P<0.05 and **P<0.01 were determined by two-tailed t-test. (E–J) H460 cells transfected with Flag-vector or Flag-p53-374*48 were treated with Doxorubicin (DOX) (E, F), Cisplatin (Cis) (G, H), or Nutlin (Nut) (I, J) for 16 h and then harvested for IB analysis with indicated antibodies and RT-qPCR analysis with primers for specific genes as indicated.

Supplementary Figure 4 p53-374*48 is predominantly present in the cytoplasm and interacts with wild type p53.

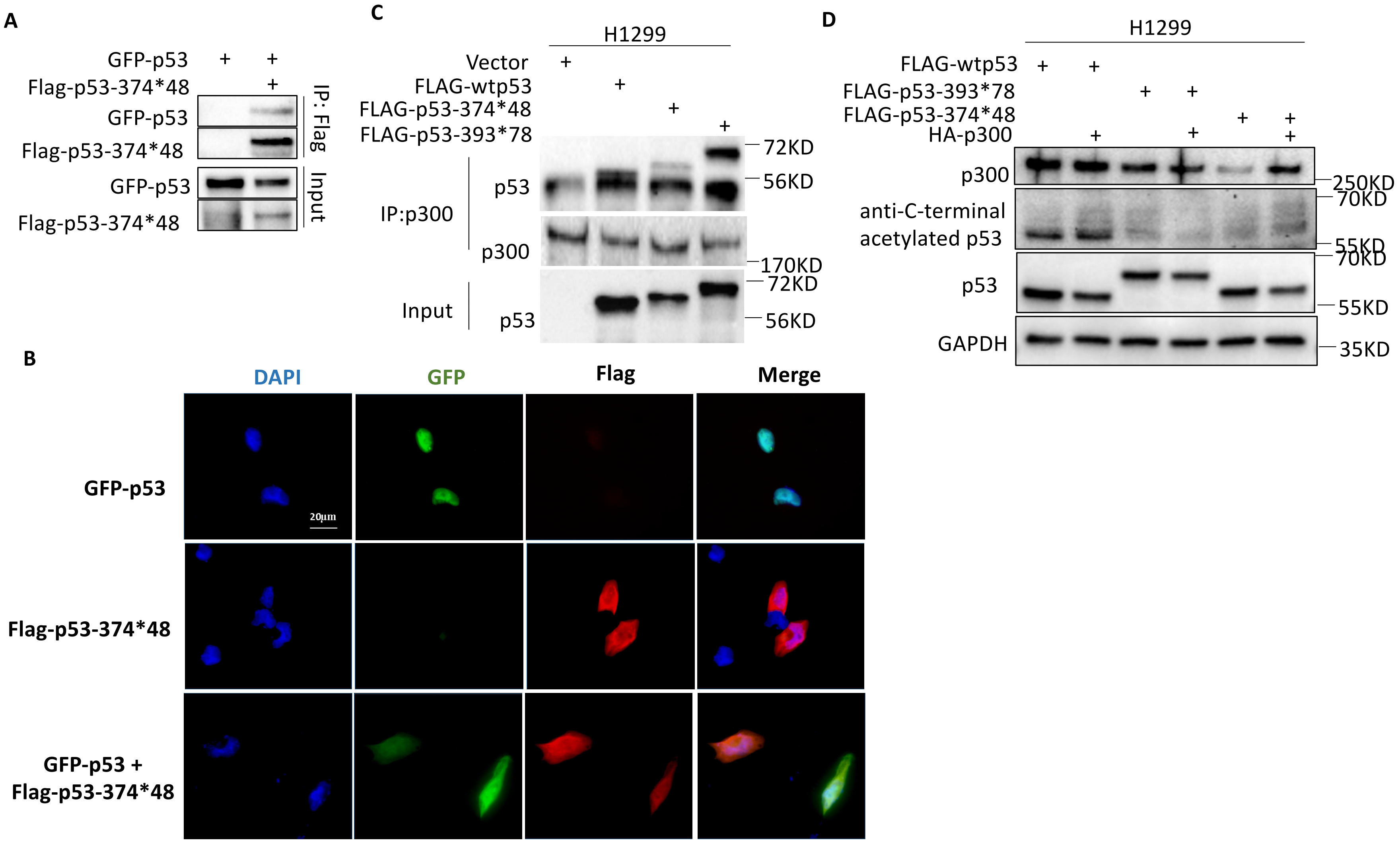

(A) H1299 cells were transfected with plasmids encoding Flag-p53-374*48 and GFPwtp53 as indicated followed by Co-IP-IB assays with indicated antibodies. (B) H1299 cells were transfected with GFP-p53 or/and Flag-p53-393*78 for 36 hrs for IF staining with the anti-Flag antibody (red), and counterstained with DAPI. Representative images with a scale bar of 20 μ m are shown here. (C) H1299 cells were transfected with plasmids encoding wt p53 or Flag-p53LCs as indicated followed by a co-IP-IB assays with indicated antibodies. (D) H1299 cells were transfected with Flag-wt p53, Flag-p53-393*78 or Flag-p53-374*48 with or without HA-p300, and harvested 48 hrs after transfection for IB analysis with indicated antibodies.

Supplementary Figure 5 p53-393*78 and p53-374*48 are not degraded by MDM2.

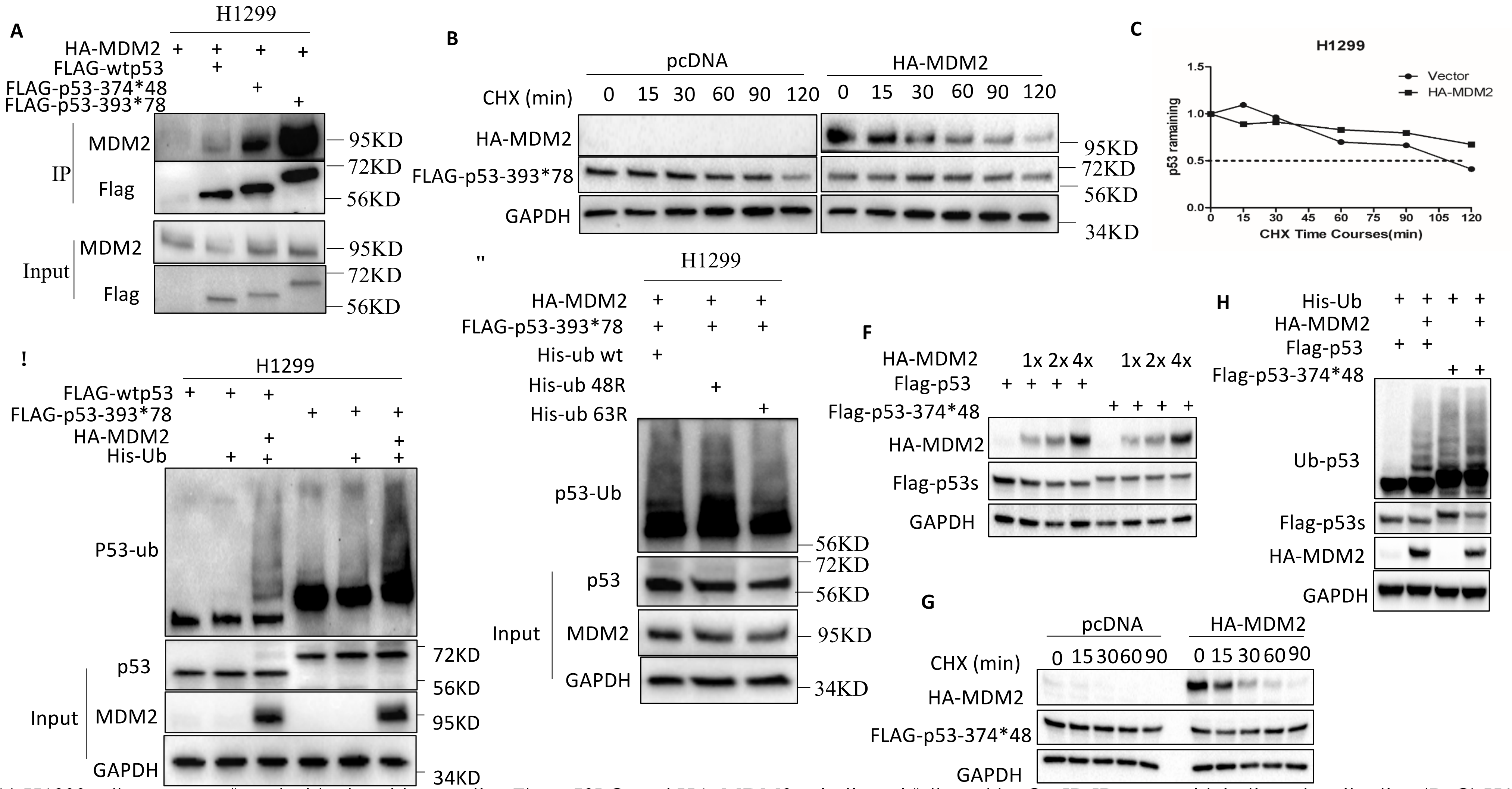

(A) H1299 cells were transfected with plasmids encoding Flag-p53LCs and HA-MDM2 as indicated followed by Co-IP-IB assays with indicated antibodies. (B, C) H1299 cells transfected with p53-393*78 in the presence or absence of HA-MDM2 for 48 h were treated with 100 μ g/ml CHX and harvested at different time points as indicated for IB analysis with indicated antibodies (B), and quantified by densitometry and plotted against time to determine p53-half-lives (C). (D) H1299 cells were transfected with plasmids encoding p53-393*78, HA-MDM2, or His-Ub as indicated, and treated with MG132 for 6 h before being harvested for an in vivo ubiquitination assay as described in the Star Methods. Bound and input proteins were detected by IB analysis with antibodies as indicated. (E) H1299 cells were transfected plasmids encoding p53-393*78, HA-MDM2, or His-Ub/48R/63R as indicated, and treated with MG132 6 h before harvest for in vivo ubiquitination assay. Bound and input proteins were

detected by immunoblotting using antibodies as indicated. (F) H1299 cells were transfected with plasmids encoding Flag-p53s and HA-MDM2 as indicated followed by IB analysis with indicated antibodies. (G) H1299 cells transfected with p53-374*48 in the presence or absence of HA-MDM2 for 48 h were treated with 100 μg/ml CHX and harvested at different time points as indicated. The p53 protein level was detected by IB analysis with antibodies as indicated. (H) H1299 cells were transfected with combinations of plasmids encoding p53s, HA-MDM2, and His-Ub as indicated, and treated with MG132 6 h before being harvested for the in vivo ubiquitination assay. Bound and input proteins were detected by IB analysis with antibodies as indicated.

Supplementary Figure 6 p53-374*48 desensitizes wt p53 bearing cancer cells to Nutlin treatment.

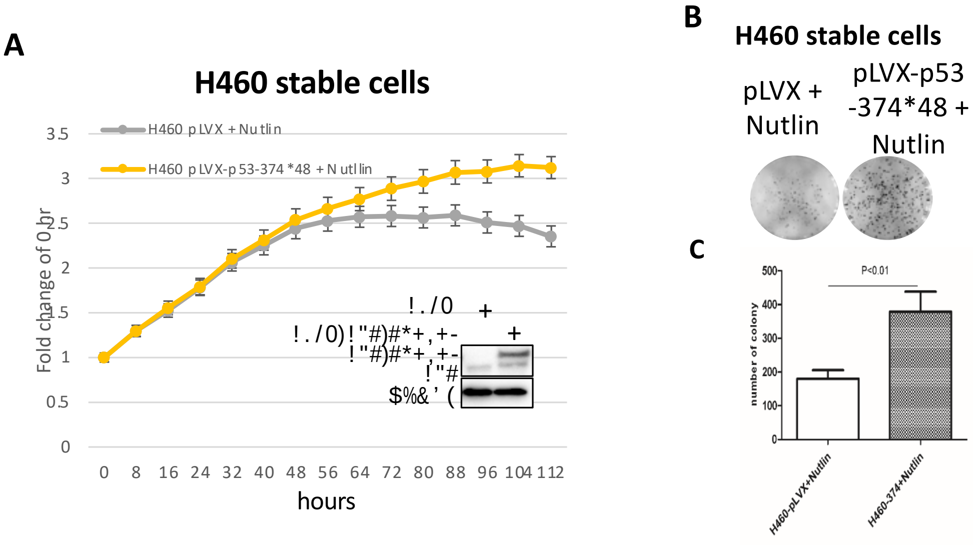

(A–C) H460 cells that stably expressed vector or p53-374*48 were treated with Nutlin (10 μm) for cell viability evaluation every 24 h by CCK-8 assay (mean ± SEM, n = 6) (A) or the colony formation assay (B, C) as described in the Star Methods. The number of colonies quantified as shown in panel C. Data were presented as mean ± SEM of triplicate experiments.*P<0.05 and **P<0.01 were determined by two-tailed t-test.
